# Drug Delivery of siRNA Therapeutics

**DOI:** 10.3390/pharmaceutics12020178

**Published:** 2020-02-20

**Authors:** Gaetano Lamberti, Anna Angela Barba

**Affiliations:** 1 Eng4Life Srl, Spin-off Accademico, Via Fiorentino, 32, 83100 Avellino, Italy; glamberti@unisa.it; 2 Dipartimento di Ingegneria Industriale; Università degli Studi di Salerno, via Giovanni Paolo II, 132 84084 Fisciano (SA), Italy; 3 Dipartimento di Farmacia; Università degli Studi di Salerno, via Giovanni Paolo II, 132 84084 Fisciano (SA), Italy

Small interfering RNA (siRNA) is a class of nucleic acid-based drugs (NABDs) able to block gene expression by interaction with mRNA before its translation. Small interfering RNAs (siRNAs) therefore present extraordinary potential due to their ability to silence the expression of disease-causing genes. Even if the mechanism of action has been successfully investigated (Nobel Prize in Physiology or Medicine 2006 to Andrew Z. Fire and Craig C. Mello “for their discovery of RNA interference – gene silencing by double-stranded RNA”) and siRNA drugs can be candidates to fight, in principle, any diseases. However, the practice of siRNA-based therapies is restricted because of relevant inconveniences. SiRNAs are negatively charged large macromolecules and this entails difficult crossing of cell membranes; they undergo rapid degradation by plasma enzymes and are easily subjected to fast hepatic/renal clearance sequestration. These features seriously hinder siRNAs’ usability in therapeutics. Currently, the scientific community focused on gene therapy research is developing studies to overcome the obstacles related to siRNA’s features.

This Special Issue of *Pharmaceutics* titled “Drug Delivery of siRNA Therapeutics” aims to present the state of the art of siRNA delivery, embracing investigation strategies of international research groups with different experiences and skills. The Special Issue will thus be devoted to presenting the current connections between experimental and in silico approaches for therapies based on siRNA delivery, accounting for all the most promising techniques based on liposomes, polymeric and inorganic nanoparticles, aptamers, chemical modification of siRNAs, and so on.

Reviews (five) and research papers (eight) constitute this Special Issue. A representative international scientific community focused on gene-therapies researches is represented by 12 different countries involving 75 scientists with multidisciplinary skills.

In the reviews, different research activities cover several disciplines of investigation mainly focused on approaches of siRNA therapies to combat several kinds of cancer in laboratory conditions and the current state of siRNA–lipid delivery systems in clinical trials.

In Marson et al., [1] studies on poly(amidoamine)-based dendrimers as attractive nanovectors for siRNA delivery into cells, were presented. In particular, an introduction to RNAi-based therapeutics and the advantages offered by dendrimers as siRNA nanocarriers were discussed. Subsequent linked studies reported in Laurini et al., [2] present the development of poly(amidoamine)-based amphiphilic dendrons—structures able to auto-organize themselves into nanosized micelles which ultimately outperform their covalent dendrimer counterparts in in vitro and in vivo gene silencing. In Barba et al., [3] the current status of siRNA-lipid delivery systems in clinical trials was addressed, offering an updated overview on the clinical goals and the next challenges of this new class of therapeutics which will soon replace traditional drugs. Farra et al., [4] focused their studies on the description of the therapeutic potential of siRNAs and polymer-/lipid-based delivery systems for ovarian cancer. After a brief description of ovarian cancer and siRNA features, they summarized the strategies employed to minimize siRNA delivery problems, the targeting strategies to ovarian cancer and the preclinical models available. They also discussed the most interesting works published in the last three years about polymer-/lipid-based materials for siRNA delivery. In Dinis Ano Bom et al., [5] attention was devoted to the use of aptamers as delivery agents of siRNA in nanoparticle formulations in cancer treatments, alone or in combination with chemotherapy.

Research papers deal with experimental new strategies to design and develop innovative suitable and effective vectors for siRNA delivery such as liposomes, dendrimers, aptamers, polymer–lipid systems, polymeric, co-polymeric and magnetic nanoparticles.

Stiina Kontturi et al., [6] aimed their studies at the development of efficient and safe administration systems devoted to the delivery of oligonucleotide-based drugs. In particular, they produced a light-triggered liposomal delivery system for oligonucleotide delivery based on a non-cationic and thermosensitive liposome with indocyanine green as a photosensitizer ingredient. Hao et al., [7] focused their studies on the combination of chemotherapeutic drugs and siRNA as an emerging modality for cancer therapy. They developed a functionalized mixed micelle-based delivery system for targeted co-delivery of methotrexate and survivin siRNA. Hattori et al., [8] presented studies on three types of cationic liposomes/siRNA complexes (siRNA lipoplexes) on gene-silencing actions in tumor cells. They used three types of cationic cholesterol derivatives to investigate an optimal formulation to achieve the best performance in terms of gene-silencing and cellular uptake effects. In Egorova et al., [9] researches on modular peptide carriers for the delivery of siRNAs to therapeutic angiogenesis inhibition were performed. In particular, the transfection properties of siRNA as polyplexes were studied in breast cancer cells and endothelial cells. Fatemian et al., [10] investigated the use of inorganic pH-dependent carbonate apatite nanoparticles to efficiently deliver various classes of therapeutics into cancer cells. Co-delivery of drugs and genetic materials (siRNAs) was studied in in vivo research. Ewe et al., [11] presented a research on the chemical modifications of polyethylenimines used to produce polymeric nanoparticles, promising structures towards the development of more efficient non-viral delivery systems. In particular, they concentrated their attention on tyrosine-modified polyethylenimines with low or very low molecular weight for siRNA delivery. Jin et al., [12] focused their work on polyethyleneimine-modified magnetic Fe_3_O_4_ nanoparticles prepared for the delivery of therapeutic siRNAs to contrast oral cancer cells’ growth. Craparo et al., [13] studied the formulation and properties of a novel protonable copolymer, based on polyaspartamide, able to form polyplex structures with siRNA to be used in antiasthmatic therapy.
